# Characterizing Illness Perception Phenotypes in Older Adults With Chronic Kidney Disease

**DOI:** 10.1093/geroni/igaa057.2817

**Published:** 2020-12-16

**Authors:** Eleanor Rivera, Karen Hirschman, Raymond Townsend

**Affiliations:** 1 University of Pennsylvania, Philadelphia, Pennsylvania, United States; 2 NewCourtland Center for Transitions and Health, Philadelphia, Pennsylvania, United States

## Abstract

An individual’s understanding of their chronic illness (illness perception) is a psychological resource that has an impact on coping and self-management behaviors. Our previous study identified illness perception phenotypes (overall patterns of illness perceptions) in a sample of older adults with heart failure, COPD, and chronic kidney disease. These phenotypes were associated with perceived self-management ability (patient activation) and recent hospitalizations. To further characterize the illness perception phenotypes we focused on older adults with chronic kidney disease, analyzing illness perception data along with potential covariates from the multi-center longitudinal Chronic Renal Insufficiency Cohort study (CRIC). Covariates include sociodemographics, disease parameters, personality type, disease knowledge, and treatment adherence. While personality type was associated with illness perception phenotype, disease knowledge and treatment adherence were not. We have also conducted qualitative analyses of in-depth interviews. These results will inform the development of a pilot intervention incorporating illness perception information into the clinical setting.

